# Construction and Characterization of a *Streptococcus suis* Serotype 2 Recombinant Expressing Enhanced Green Fluorescent Protein

**DOI:** 10.1371/journal.pone.0039697

**Published:** 2012-07-20

**Authors:** Tao Chen, Qin Huang, Zhaolong Li, Wei Zhang, Chengping Lu, Huochun Yao

**Affiliations:** Key Lab of Animal Bacteriology, Ministry of Agriculture, Nanjing Agricultural University, Nanjing, China; University of Kansas Medical Center, United States of America

## Abstract

*Streptococcus suis* serotype 2 (*S. suis* 2) is an important pathogen, responsible for diverse diseases in swine and humans. To obtain a *S. suis 2* strain that can be tracked *in vitro* and *in vivo*, we constructed the Egfp-HA9801 recombinant *S. suis* 2 strain with *egfp* and *spc^r^* genes inserted via homologous recombination. To assess the effects of the *egfp* and *spc^r^* genes in HA9801, the biochemical characteristics, growth features and virulence in Balb/C mice were compared between the recombinant and the parent HA9801 strain. We detected the EGFP expression from Egfp-HA9801 by epifluorescence microscopy. The results showed that the biochemical characterization and growth features of the Egfp-HA9801 recombinant were highly similar to that of the parent HA9801. We did not find significant differences in lethality (50% lethal dose), morbidity and mortality between the two strains. Furthermore, the bacterial counts in each various tissues of Egfp-HA9801-infected mice displayed similar dynamic compared with the HA9801-infected mice. Our results also showed that the Egfp-HA9801 cells grown at 37°C for 36 h displayed greater green fluorescence signals than the cells grown at 28°C for 36 h and 37°C for 24 h. The fluorescence in the tissue cryosections of Egfp-HA9801-injected mice was also stronger than that of the HA9801 group. Together, these results indicate that the *egfp* and *spc^r^* insertions into the Egfp-HA9801 recombinant did not significantly change the virulence when compared with HA980, and this EGFP labeled strain can be used for future *S. suis* 2 pathogenesis research.

## Introduction

*Streptococcus suis* is a Gram-positive coccus that is considered an important swine pathogen and a zoonotic agent causing meningitis, arthritis, septicemia and even sudden death in swine and humans [1], [2]. Thus far, 35 different serotypes of *S. suis* have been described. Serotype 2 is the most virulent and the most frequently isolated from both diseased swine and humans [3]. *S. suis* 2 is widely distributed around the world, and not only bring large losses to the pig industry, but it also threatens the public health. In china, *S. suis* 2 was first isolated in Guangdong Province and did not attract wide attention as the cause of an emerging infectious disease until an outbreak occurred in Sichuan province, China where many people were infected, and some of them died in 1998 [4]. In July, 2005, the largest outbreak of human *S. suis* infection occurred in Sichuan province, China, where 204 people were infected, and 38 of them died [5]. The repeated intensive outbreaks of human *S. suis* infection have raised great public concern worldwide regarding the pathogenic mechanisms of this bacteria.

There have been increasing numbers of studies on virulence factors and pathogenesis of *S. suis* 2, and several molecules have been implicated as virulence factors, including the capsule polysaccharide (CPS) [6], a hemolysin (suilysin) [7], [8], a 136-kDa muramidase-released protein (MRP), and a 110-kDa extracellular factor (EF) protein[9]. In addition, it was shown recently that a fibronectin and fibrinogen-binding protein plays an important role in the colonization of affected organs after experimental infection [10]. *S. suis* 2 is usually transmitted by secretions of the oral or nasal mucosa and colonizes the palatine tonsil of both clinically ill and apparently healthy pigs [1]. The entry of *S.suis* into blood from tonsils, uptake by and travel within monocytes and entry into the brain as part of the normal circulation of monocytes play important roles in pathogenesis [11]. Hence, the outcome of the interaction between bacteria and blood phagocytes is considered a key step in the pathogenesis of *S. suis* infections [12].

Studies using chemi-fluorescent dyes (fluorochrome) for labeling bacteria have been described [13], [14]. However, because of non-specificity of the chemicals, the extrinsic labeling compounds may leak from the bacteria to phagocytes. Additionally, these approaches can be of limited value for following bacterial pathogens within live host cells. Moreover, the bacteria may be damaged by the dye and bacterial division dilutes the fluorescence signal during infection [15].

Green fluorescent protein (GFP) from jellyfish *Aequoria victoria* is a fluorescent marker that is used for studying the localization, structure, and dynamics of living cells. The fluorescent protein is soluble in a wide variety of species, can be monitored non-invasively by external illumination, and needs no external substrates [16]. The *gfp* gene, as a potential visible marker for tracking lactic acid bacteria, has been expressed by placement downstream of the constitutive *Lactococcus lactis* P32 promoter [17]. GFP has also been expressed in a number of different bacterial species, including *Escherichia coli*, *L. lactis* and *Streptococcus gordonii* [18]. Expression of GFP did not alter bacterial interactions with host cells, and bacteria producing GFP could be visualized within live mammalian cells [15]. Furthermore, a number of GFP mutant forms have been developed that exhibit enhanced fluorescence emission or altered fluorescence spectra such as enhanced GFP (EGFP).

In this study, we constructed the *S. suis* 2 Egfp-HA9801 recombinant which expresses the EGFP protein. Egfp-HA9801 was labeled successfully by inserting the *egfp* and *spc^r^* genes into the SS2 HA9801 genome through homologous recombination. The EGFP labeled Egfp-HA9801 was then compared with the parent strain HA9801 by analysis of biochemical characteristics, growth features, experimental infections and EGFP expression.

## Materials and Methods

### i. Ethics Statement

All animals used in this study, and animal experiments, were approved by Department of Science and Technology of Jiangsu Province.

### ii. Bacterial Strains, Plasmids, and Culture Conditions

The bacterial strains and plasmids used in this study are listed in Table 1. *S. suis* cells were grown on agar plates made with Todd–Hewitt (TH) broth containing 2% agar (Bacto) or in liquid cultures of TH broth. *E. coli* cells were grown on Luria–Bertani (LB) agar plates or in liquid LB broth. When necessary, antibiotics were added to the plate or broth at the following concentrations: spectinomycin (spc), 100 mg/ml for *S. suis* 2, 50 mg/ml for *E. coli* TOP 10 strain.

**Table 1:** Bacterial strains, and plasmids used in this study.

Strains, plasmids and primers	Description	Reference or source
Strains		
* S. suis* HA9801	Virulent strain of SS2Virulent strain of SS2 isolated from dead pig	Collected in our laboratory
Egfp-HA9801	The insertion mutant of *egfp* and *spc^r^* with background of HA9801, Spc^r^	In this study
E. coli TOP 10	Cloning host for maintaining the recombinant plasmids	Collected in our laboratory
Plasmids		
pEVP3	A suicide vector, Cm^r^	[19]
pESEB	A recombinant vector with the background of HA9801,designed for insertion of *egfp* gene;Spc^r^,Cm^r^	In this study
pEGFP-N1	A green-fluorescence protein vector	Clontech, Palo Alto, CA
pSET2	A plasmid containing Spc^r^ gene	[20]

Cm^r^, chloromycetin resistant; Spc^r^, spectinomycin resistant.

### iii. Construction of the *S. suis* 2 Recombinant Egfp-HA9801

**Figure 1:**
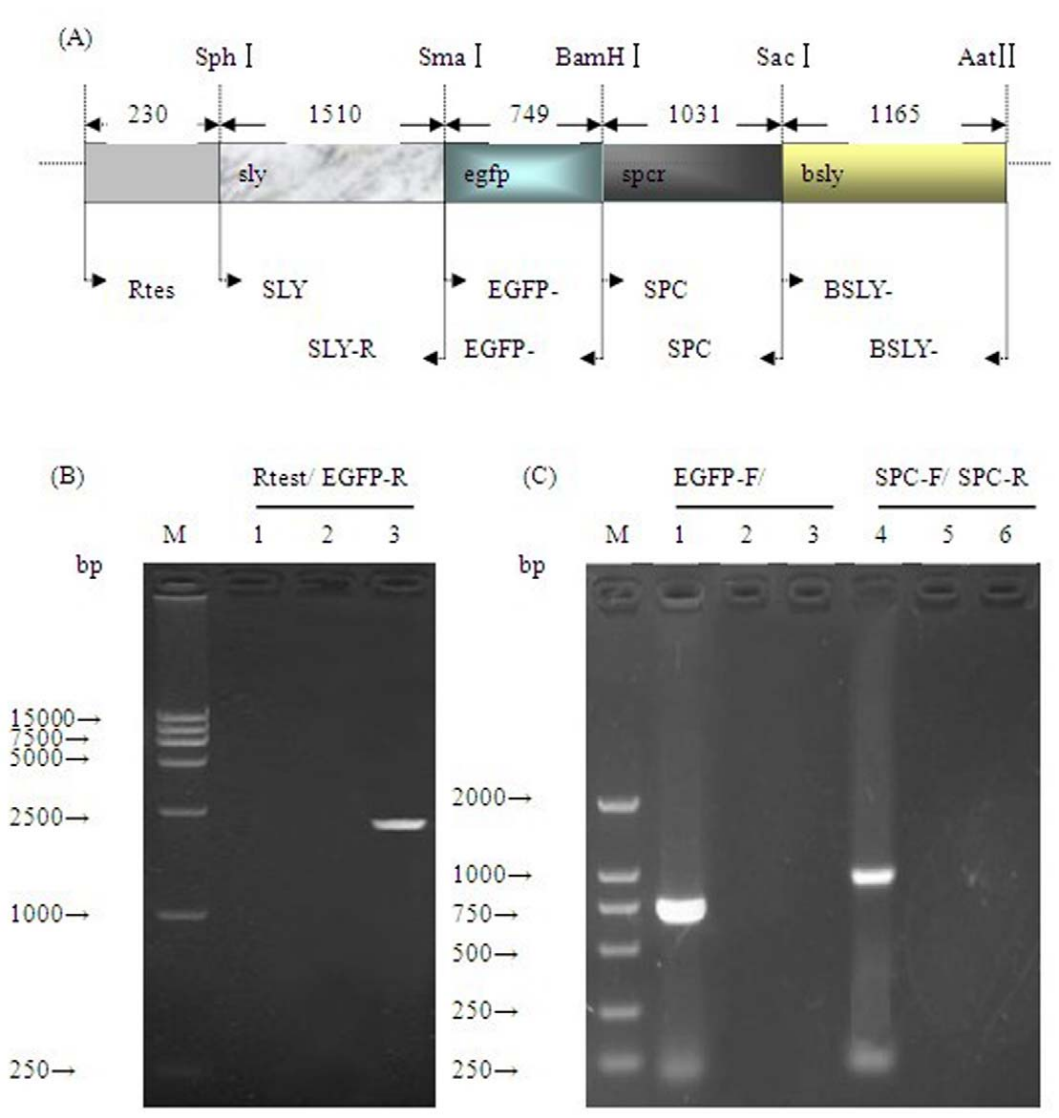
Genomic organization of the double crossover recombination locus in *S. suis* 2 Egfp-HA9801 and confirmation analysis of the recombinant strain Egfp-HA9801. (A) Genomic organization of the double crossover recombination locus and its flanking genes in *S. suis* 2 Egfp-HA9801. Dashes above the gene indicate restriction sites. The numbers above the gene and between the solid arrows indicate the size (bps) of the known gene fragments. The different color boxes represent *sly, egfp,spc^r^* and *bsly* genes. The location of the primers used in PCR and RT-PCR detection are indicated by inverted arrowheads. (B) PCR analysis of the Egfp-HA9801 recombinant strain. The PCR primer combinations are shown over the lanes. Genomic DNA from the parent strain HA9801 (lane 2) and Egfp-HA9801 recombinant (lane 2) were used as templates. Lane 1 is the negative control. The 15 kb DNA ladder marker is shown to the left (M). (C) RT- PCR analysis of the Egfp-HA9801 recombinant strain. The primer combinations used in RT-PCR are shown over the lanes. Total RNA extracted from mid-exponential-phase cultures of the following strains were used as templates: parent strain HA9801 (lane 2, 5) and Egfp-HA9801 recombinant (lane 1, 4). Lane 3 and lane 6 are negative controls. The 15 kb DNA ladder marker is shown to the left (M). Theoretical size (bp) of each of the PCR and RT-PCR products generated with the primer combinations are shown in (A).

The recombinant plasmid (pESEB) was constructed by cloning four flanking gene fragments into the pEVP3 plasmid. First, the flanking DNA sequences to *sly* and *bsly* were amplified from the chromosomal DNA of *S. suis* 2 HA9801 using PCR with two pairs of specific primers (SLY-F/SLY-R and BSLY-F/BSLY-R) carrying *Sph* I/*Sma* I and *Sac*I/*Aat* II restriction enzyme sites, respectively (Table 2). Following digestion with the corresponding restriction enzymes, the DNA fragments were cloned into a pEVP3 vector directionally. Then, the *egfp* gene (from pEGFP-N1) was inserted at the *Sma* I/*BamH* I sites, and the *spc^r^* gene cassette (from pSET2) was inserted at the *BamH* I/*Sac* I sites (Fig. 1 A). Finally, the resulting recombinant plasmid (pESEB) was confirmed by PCR and restriction enzyme digestion. To obtain the recombinant strain, the pESEB plasmid was introduced into *S. suis* HA9801 competent cells by electroporation. For preparation of *S. suis* competent cells, wild-type *S. suis* was grown in TH broth containing 40 mmol/L D-L- threonine to OD600 of 0.4 at 37°Cand washed twice with sterile water after incubation on ice for 30 min. Subsequently, the cells were resuspended in a mixture containing 15% glycerol and 0.3 mol/L sucrose and stored at −75°C. The electroporation settings were: resistance 250 Ω; voltage: 2.3 kV (cuvette gap: 2.0 mm) and time constant:4.9 ms. The transformed cells were incubated at 37°C for 120 min and then plated on TH agar plates containing 200 µg/ml spectinomycin and incubated for 48 h at 37°C. A single colony was picked and inoculated in 5 ml TH Broth containing 200 µg/ml spectinomycin and incubated overnight at 37°C. The resulting recombinant strain was verified by PCR and RT-PCR amplification and by direct DNA sequencing of the recombination sites using genomic DNA preparations of the recombinant strains.

**Table 2:** Primers used for PCR amplification and detection.

Primers	Sequence of primers (5′–3′′)	Restriction sites	Functions
SLY-F	ACAT**GCATGC**ATGAGAAAAAGTTCGCAC	*Sph* I	sly gene(1510 bp)
SLY-R	TCC**CCCGGG**CTCTATCACCTCATCCGC	*Sma* I	
BSLY-F	C**GAGCTC**TCTGGCAATGTATTAT	*Sac* I	bsly gene,downstream fragment of sly (1165 bp)
BSLY-R	GGG**GACGTC**CTTATCAGCAAAAAGA	*Aat* II	
SPC-F	**GGATCC** GTTCGTGAATACATGTTAT	*BamH* I	Spc^R^ gene cassette(1031 bp)
SPC-R	**GAGCTC** GTTTTCAAAATCTGATTA	*Sac* I^+^	
EGFP-F	TCC**CCCGGG**ATGGTGAGCAAGGGC	*Sma* I^-^	egfp gene(749 bp)
EGFP-R	CGC**GGATCC**TTACTTGTACAGCTCGT	*BamH* I	
Rtest	AGCTCGTTGCCCTTGT		

The restriction sites are in bold.

### iv. Biochemical Characterization of the Recombinant *S. suis* 2 Egfp-HA9801

Single colonies from the parent HA9801 and Egfp-HA9801 strains, grown on TH broth agar containing 5% (v/v) sheep blood at 37°C for 24 h, were picked and inoculated into fresh 5 ml THB medium. Then the parent HA9801 and Egfp-HA9801 cells were continually passaged 20 times in 5 ml THB medium, and the biochemical characterizations of the F1, F10 and F20 passage were performed using the automatic biochemistry analyzer (bioMerieux VITEK 2 System).

### v. Growth of the Recombinant *S. suis* 2 Egfp-HA9801

Inoculations of 1.0×10^9 ^CFU bacteria, equal to 1% (v/v) of 100 ml of THB medium, were incubated in fresh THB at 37°C for 18 h. During the 18 h period, aliquots of 2 ml of cultures were removed every hour to measure the optical density at 600 (OD600) indicating the bacterial concentration.

### vi. Experimental Infections of Balb/C Mice

A total of 155 female Balb/C mice at 6 weeks of age (Experimental Animal Center of Yangzhou University) were included in the study. All experiments involving mice were repeated twice and were conducted in accordance with the International Guiding Principles for Biomedical Research Involving Animals–1985. The mice were acclimatized to standard laboratory conditions of 12-h light/12-h dark cycle with free access to rodent chow and water. For all groups, mice showing severe clinical signs and considered moribund were humanely euthanized. Bacterial working cultures were prepared as previously described [21]. Stationary phase bacteria were washed twice in PBS (pH 7.4). Bacterial pellets were then resuspended and serially diluted in THB to appropriate concentrations. The final suspensions of the inoculum for experimental infection were plated onto THB agar to accurately determine the titer and recorded as colony forming units (CFU)/ml.

### vii. Determination of the 50% Lethal Dose (LD50)

Six-week-old female Balb/C mice (six mice in each group) were infected by intraperitoneal (IP) injection with 500 µl of either the HA9801 parent or Egfp-HA9801 recombinant with the dilutions ranging from 1.2×10^8^ to 6.0×10^8 ^CFU in THB. Mice infected with sterile THB were used as controls. The 50% lethal doses (LD50) of both strains were calculated according to the Karber method [22].

### viii. Mouse Survival and Mortality Studies

A total of 30 female Balb/C 6-week-old mice were infected by IP injection with 1 ml of either HA9801 parent or the Egfp-HA9801 recombinant at approximately 6.0×10^8 ^CFU in THB. Mice infected with sterile THB were used as controls. The mortality and clinical signs of infection such as depression, swollen eyes, rough hair coat, lethargy, and neurological signs were recorded daily post-infection (p.i.) over a 7-day (d) period as previously described [23].

### ix. Determination of Viable Bacteria in Organs

A total of 50 female Balb/C 6-week-old mice were infected by IP injection with 500 µl of either HA9801 parent or the Egfp-HA9801 recombinant at approximately 6.0×10^8 ^CFU in THB. Mice infected with sterile THB were used as controls. At each designated time, three infected and one non-infected mice were sacrificed by cervical dislocation. The presence of HA9801 or the Egfp-HA9801 in blood (1 ml) and homogenized organ (0.05 g/organ) samples was determined by plating on THB agar plates.

### x. Epifluorescence Microscopy

Egfp-HA9801 and HA9801 cells in liquid culture were washed once with 0.1 M PBS (pH7.4) and resuspended in 30% glycerol. Fluorescence was observed by applying 4 µl of the cell suspension on a microscope slide followed by examination using a Carl Zeiss Observer Z1 inverted epifluorescence microscope. The objective was a 40×10.6 LD Plan-Neofluar and a 100×1.40 Oil Plan-Apochromat. The excitation source was a 100 W HBO bulb, and digital images were captured with a 14 bit cooled standard-scan charge coupled-device camera (AxioCam MR3; Carl Zeiss) with resolution set at 1388×1040. The charge-coupled-device camera was controlled by the AxioVision software (version Rel.4.6, Carl Zeiss), and a FITC filter set (exciter: HQ480/40; emitter: HQ 535/50) was used for the excitation and detection of EGFP. The tissue cryosections from the dead mice, which were IP inoculated with 6.0×10^8^ CFU Egfp-HA9801 or HA9801 bacteria, were also checked immediately by epifluorescence microscopy.

### xi. Statistical Analysis

Unless otherwise specified, all data are expressed as means ± SD and analyzed by a two-tailed, unpaired Student’s t-test. A *P* value <0.05 is considered statistically significant.

## Results

### i. Construction and Characterization of the *S. suis* 2 Egfp-HA9801 Recombinant

The recombinant strain was first screened on THB agar plates under the selective pressure of spectinomycin and then confirmed by PCR (Fig. 1 B) and RT-PCR (Fig. 1 C). The direct DNA sequencing result of the recombination sites also indicated that the *egfp* and *spc^r^* gene had been inserted into HA9801 genome successfully.

### ii. Biochemical Characterization of Egfp-HA9801

The results of the biochemical parameters of Egfp-HA9801 are shown in Table 3. The parameters of Egfp-HA9801 were the same as that of the parent HA9801 strain, except for arginine dihydrolase 1, D-Galactose, and pyroglutamate enzyme aromatic amine. There was also no difference between the different passages of Egfp-HA9801. These results indicate that the biological characteristics of Egfp-HA9801 were stable and similar to HA9801, and had not been influenced by inserting the *egfp* and *spc^r^* genes into the *S. suis* 2 genome.

**Table 3:** Biochemical characteristics of the *S. suis* 2 (SS2) parent strain and recombinant obtained using a automatic biochemistry analyzer.

	SS2 HA9801 parent strain			SS2 Egfp-HA9801 recombinant		
Parameters	F1	F10	F20	F1	F10	F20
Amygdalin	−	−	−	−	−	−
Phosphatidylinositol phospholipase C	−	−	−	−	−	−
D-Xylose	−	−	−	−	−	−
Arginine dihydrolase 1	+	(+)	+	(−)	−	−
β-D-galactosidase	−	−	−	−	−	−
α-glucosidase	+	+	+	+	+	+
Ala-Phe-Pro aromatic amines enzyme	+	+	+	+	+	+
β-Gal-pyran glycosidase	−	−	−	−	−	−
α-Mannosidase	−	−	−	−	−	−
β-glucuronidase	+	(+)	(+)	+	+	+
α-galactosidase	+	+	+	+	+	+
Pyroglutamate enzyme aromatic amine	(−)	−	(−)	(+)	+	+
β-D-Glucuronidase	−	−	−	−	−	−
Enzyme alanine aromatic amines	+	+	+	+	+	+
Tyrosinase enzyme aromatic amine	+	+	+	+	+	+
D-galactose	+	+	+	−	−	(−)
Lactose	+	+	+	+	+	+
N-acetyl-D-glucosamine	+	+	(+)	+	+	+
D-Maltose	+	+	+	+	+	+
Bacitracin tolerance	−	−	−	−	−	−
Methyl-B-D-glucose pyran-glucoside	+	+	+	+	+	+
Sucrose	+	+	+	+	+	+

+: Positive; −: Negative; (): Results Weak.

### iii. Growth Features of Egfp-HA9801

**Figure 2:**
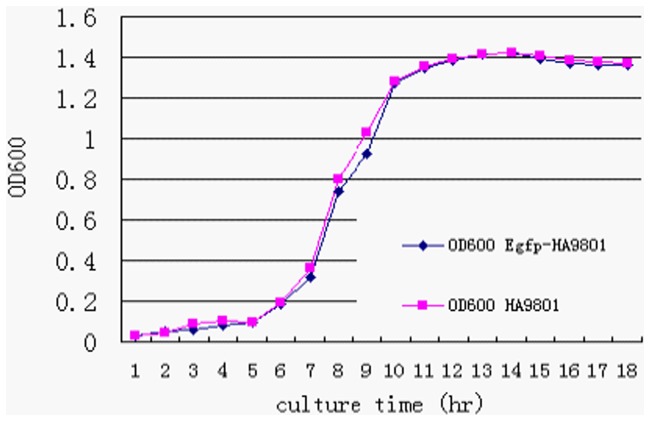
Growth curve of Egfp-HA9801 and parent HA9801. Both Egfp-HA9801 and HA9801 were incubated in 100 ml of fresh THB at 37°C for 18 h. During the 18 h period, aliquots of 2 ml of cultures were used to monitor the bacterial concentration every hour. No significant differences were found between the two strains throughout the experiment (*P*>0.05).

Cultures of both Egfp-HA9801 and the parent HA9801 strains at different times were used to measure their bacterial concentration (Fig. 2). The growth curve of Egfp-HA9801 was similar to that of HA9801.

### iv. Experimental Infections of Balb/C Mice

To study the effect of *egfp* and *spc^r^* insertions on the pathogenesis of *S. suis* 2, the virulence of the Egfp-HA9801 recombinant and parent HA9801 was assessed in a Balb/C mouse infection model. Results of the trial showed that the LD50 of Egfp-HA9801 and HA9801 were 3.200×10^8 ^CFU and 3.259×10^8^CFU, respectively (Table 4), indicating that there were no significant differences between the two strains (*P*>0.05).

**Table 4:** LD50 of *S. suis* type 2 HA9801strains and recombinant SS2 Egfp-HA9801 strains in Balb/C mice.

Strain	Infection dose (CFU)	Amount of mouse	Mortality	LD50 (CFU)
	6.0×10^8^	6	6/6	
	4.8×10^8^	6	5/6	
HA9801	3.6×10^8^	6	3/6	3.259×10^8^
	2.4×10^8^	6	2/6	
	1.2×10^8^	6	0/6	
				
	6.0×10^8^	6	6/6	
	4.8×10^8^	6	5/6	
Egfp-HA9801	3.6×10^8^	6	4/6	3.200×10^8^
	2.4×10^8^	6	1/6	
	1.2×10^8^	6	0/6	
				
Blank control		6	0/6	

The LD50 of both strains were calculated according to the Karber method.

**Figure 3:**
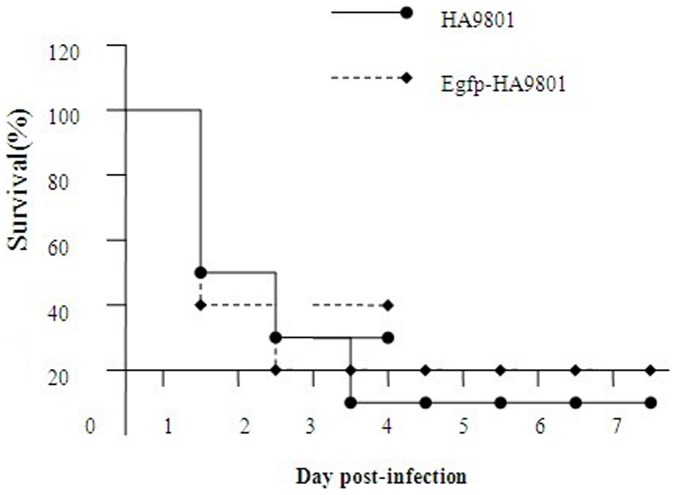
Survival curves for Balb/C mice infected with Egfp-HA9801 and HA9801strains. Six-week-old Balb/C mice were inoculated i.p. with 6.0×10^8 ^CFU bacteria; mice survival was monitored over a 7-day period. Data are expressed as mean percentage of live animals in each group (n = 10).

During the course of infection, all the mice infected with HA9801 at approximately 6.0×10^8 ^CFU showed clear clinical signs, such as depression, swollen eyes, weakness and prostration post-inoculation. Five mice died in this group during the 24 h post-inoculation. From day 4 p.i., surviving mice in the HA9801 group developed clinical signs associated with *S. suis* 2 meningitis such as hyperexcitation, opisthotonus, bending of the head and walking in circles. Meanwhile, mice infected with Egfp-HA9801 had an 80% mortality rate, and all of them showed clear clinical signs (Table 5 and Fig. 3). All the results indicated that no significant differences were found between the Egfp-HA9801 group and the HA9801 group.

**Table 5:** Virulence of the *S. suis* parent strain HA9801 and Egfp-HA9801 recombinant in Balb/C mice.

	No. of Balb/C mice	Morbidity^a^ (%)	Mortality^b^ (%)
HA9801	10	100	90
Egfp-HA9801	10	100	80
Blank control	10	0	0

These measurements were performed over a period of 7-day post-infection.

a Percentage of mice with clinical symptoms.

b Percentage of mice that died due to infection.

**Figure 4:**
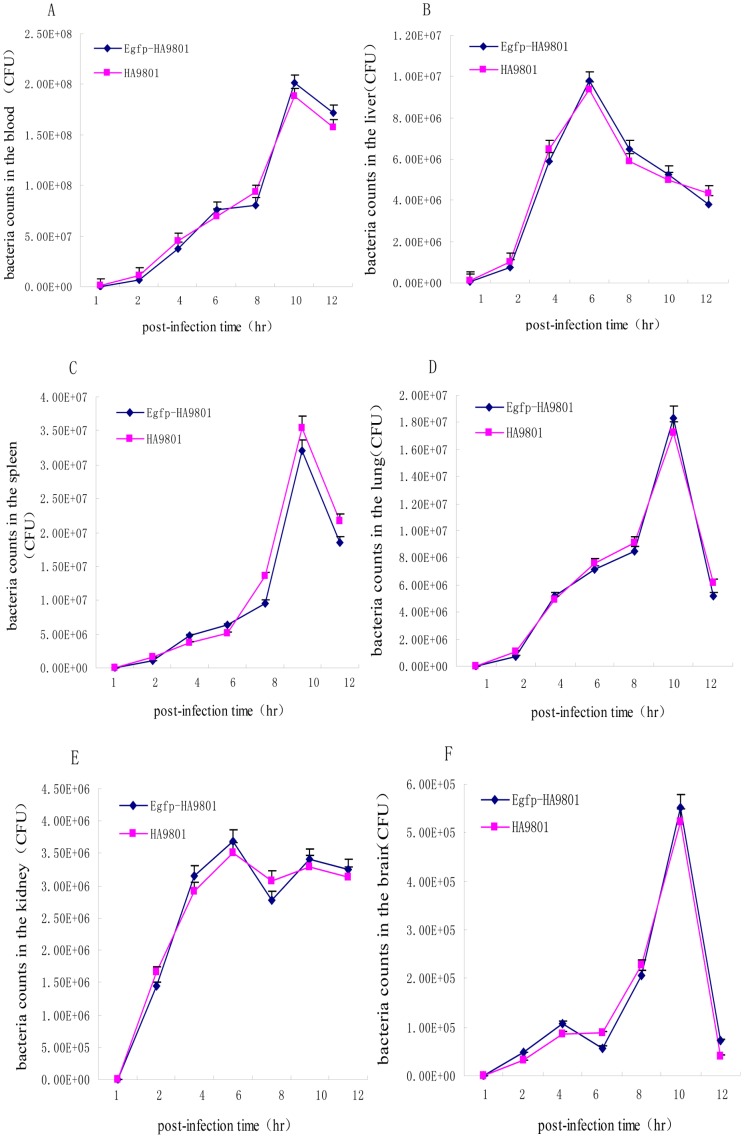
Bacterial distribution in different organs from mice infected i.p. with HA9801 parent and Egfp-HA9801 recombinant strains. Bacterial loads in the blood (A) are expressed as CFU/ml, and in the liver (B), spleen(C), lung (D), kidney (E) and brain (F) as CFU/0.05 g of tissue. Results are expressed as mean ± SEM of at least three infected mice per p.i. time point. No significant differences were found between the two strains throughout the experiment (*P*>0.05).

To further investigate the virulence of the Egfp-HA9801 recombinant, *in vivo* colonization experiments were carried out. According to the results of LD50 assessment, mice were IP inoculated with about 6.0×10^8 ^CFU in 1 ml THB of the recombinant or parental strains. The live bacteria were recovered from the lung, liver, kidney, spleen, brain and blood at each designated timepoint. As shown in Fig. 4, bacterial counts from each specific tissue of Egfp-HA9801-infected mice were not significantly different from that of the HA9801-infected mice (*P*>0.05). These results suggested that the colonization of Egfp-HA9801 had not been obviously influenced by inserting *egfp* and *spc^r^* gene into the *S. suis* 2 genome. Together with the above results, this shows that Egfp-HA9801 was very similar with HA9801 in terms of virulence, presentation of symptoms and infection process. Therefore, the recombinant strain can be used as an EGFP labeled strain for SS2 pathogenesis research under natural conditions.

### v. Expression of EGFP in *S. suis* Recombinant Egfp-HA9801 as Detected by Epifluorescence Microscopy

**Figure 5:**
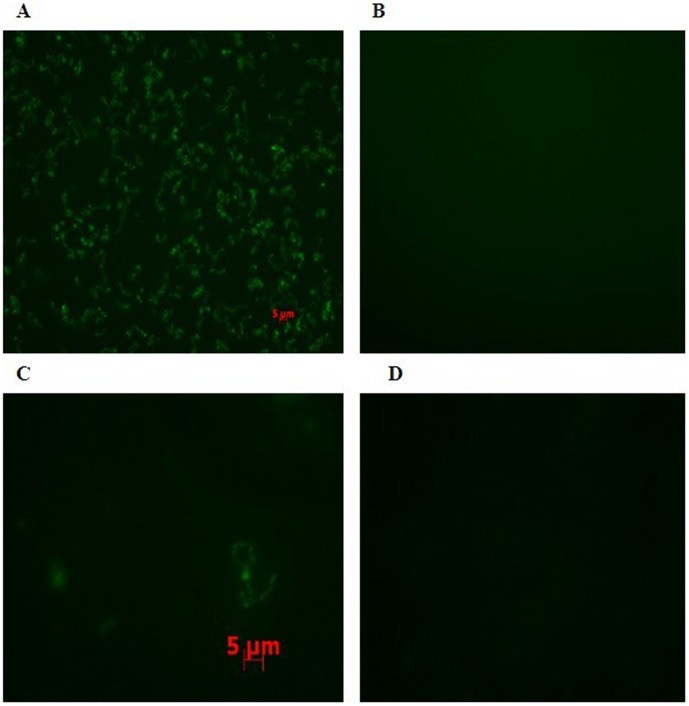
Epifluorescence microscopy analysis of bacteria cultured *in vitro*. A: Egfp-HA9801(10×40); B: HA9801(10×40); C: Egfp-HA9801(10×100); D: HA9801(10×100).

**Figure 6:**
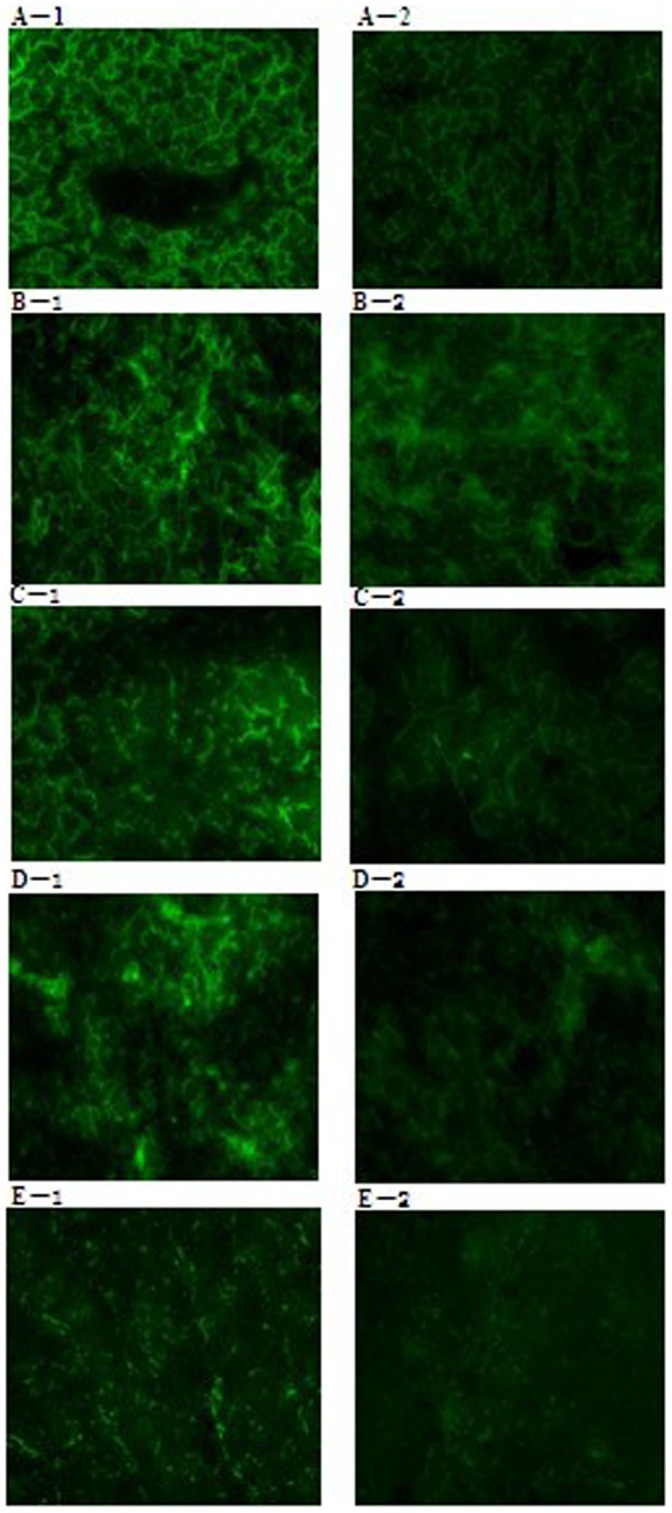
Epifluorescence microscopy analysis of the cryosections of the tissues obtained from the mice infected with Egfp-HA9801 or HA9801. The cryosections of liver (A-1), lung (B-1), kidney (C-1), spleen (D-1) and brain (E-1) from the Egfp-HA9801-infected mice were detected under epifluorescent microscope and the FITC images are shown in the left column. The FITC images of the cryosections of liver (A-2), lung (B-2), kidney (C-2), spleen (D-2) and brain (E-2) from the HA9801-infected mice, are showed in the right column.

*S. suis* recombinant Egfp-HA9801 cells were clearly fluorescent under the epifluorescent microscope, whereas no fluorescent signals from the HA9801 cells were observed (Fig. 5). In particular, the Egfp-HA9801 cells which were grown at 37°C for 36 h showed brighter fluorescent signals than the cells grown at 28°C for 36 h and 37°C for 24 h. Furthermore, the fluorescence of tissue cryosections of the Egfp-HA9801-injected mice was stronger than that of the HA9801 group (Fig. 6).

## Discussion

*S. suis* 2 is considered as one of the most important swine pathogens and is an emerging, life-threatening zoonotic agent in both pigs and humans. Although a series of virulence factors associated with *S. suis* 2 were identified recently [6], [24], the specific function of these virulence factors in the pathogenicity of *S. suis* 2 is still unknown, and the pathogenesis of the infection caused by *S. suis* is still poorly understood.

In this study, the EGFP labeled recombinant Egfp-HA9801 was constructed via homologous recombination and the impact of *egfp* and *spc^r^* gene insertion on biochemical characteristics and virulence of *S. suis* 2 was assessed. The results of this study demonstrated that the EGFP labeled Egfp-HA9801 is a promising and useful tool for the study of *S. suis* 2 pathogenesis both *in vitro* and *in vivo*.

The target organism can be labeled with GFP through plasmid-based *gfp* vectors or chromosomal marker. Although plasmid-based *gfp* vectors have been used in eukaryotic systems, and some *gfp*-broad host-range plasmids have been successfully used to label certain species of bacteria, there are two limitations to applying these vectors for bacterial strains used in environmental studies. First, due to concerns about plasmid stability under natural environmental conditions, bacterial strains used in pathogenesis studies should be chromosomally marked with a single copy of the *gfp* gene to maximize genetic stability as well as reduce the risk of transfer of the genetic marker to other microorganisms. The second limitation is the sensitivity required for detection of individual cells containing a single copy of the *gfp* marker. To circumvent these limitations, a Tn10- [25] and several Tn5-based [26], [27] transposon suicide gene delivery vectors have been developed. They have been used to mark various Gram-negative bacteria including, *Agrobacterium tumefaciens*, *Alcaligenes eutrophus*, *Moraxella sp*., and *Vibrio sp*. However, there have not been any studies on Gram-positive bacteria chromosomally engineered with a *gfp* genetic marker. In this study, the SS2 Egfp-HA9801 recombinant was chromosomally marked via homologous recombination, and the biochemical characterization and growth features of the Egfp-HA9801 recombinant were extremely similar to that of the parent HA9801. In the present work, we did not find significant differences in LD50, morbidity and mortality between the two strains, indicating that the insertion of *egfp* and *spc^r^* had no effect on bacterial virulence. Furthermore, the bacterial counts in each tissue of Egfp-HA9801-infected mice displayed similar dynamic variations compared with the HA9801-infected mice. These findings suggest that the *egfp* and *spc^r^* insertion did not render any significant changes in the Egfp-HA9801 recombinant when compared with HA9801. Therefore, the recombinant can be used as EGFP labeled strain for SS2 pathogenesis research under natural conditions.

Overall, the expression of GFP protein is stable, but GFP protein folding is thermosensitive. Moreover, the chromophore formation requires sufficient time; hence, the principal limiting factors of GFP expression include environmental temperature and expression time [16], [28]. Jin Zou *et al*. [29] reported that 72 h of expression at 30°C is appropriate for chromophore formation of the GFP variants in HeLa cells. Surprisingly, the same GFP variants could not be detected with either the fluorescence spectrophotometer or fluorescence microscope in HeLa cells when grown at 37°C after transfection. For *S.suis* 2 SX332 cells, GFP expression peaked at 48 h and 30°C [30]. Our results showed that the Egfp-HA9801 cells grown at 37°C for 36 h showed more obvious green fluorescence than the cells grown for other culture temperatures and time periods, indicating that 36 h of expression at 37°C is optimal for expression of EGFP in our recombinant strain. One possible reason is that the expression of GFP at 37°C proceeds too fast to process the necessary post-translational modification in the other GFP mutant strains but this temperature is appropriate for EGFP. Furthermore, the labeling method based on chromosomal marking may affect the optimum condition of GFP expression.

In this study, the fluorescence of tissue cryosections of Egfp-HA9801-innoculated mice was stronger than that of the HA9801 group, but the green fluorescence of the bacteria in the cryosections were not clearly seen because of the high fluorescence background of the tissue. Autofluorescence is often seen in many species and is due to various factors, including metabolites and structural components. However, the emission and excitation characteristics of autofluorescence are very different from GFP; hence, further optimization of standard filter sets may simply be made to reduce or eliminate the autofluorescence in the tissues [31]. Schnell *et al.* [32] studied the effect of chemical treatments on tissue sections from monkey, rat, and human neural tissue. They revealed that lipofuscin-like autofluorescence in these tissues could be significantly reduced or eliminated by treatment with 1–10 mmol/L CuSO_4_ in a 50 mmol/L ammonium acetate buffer (pH 5) or 1% Sudan black B in 70% ethanol. Further optimization studies are needed to reduce or eliminate the autofluorescence in the tissues.

In conclusion, our study demonstrated, for the first time, that the Egfp-HA9801 recombinant, labeled with *egfp* gene basing on chromosomal marking, is a promising and useful tool for the study of *S. su*is 2 pathogenesis both *in vitro* and *in vivo*.
